# Supplementary material for: Causal Relationship between Aging and Anorexia Nervosa: A White-Matter-Microstructure-Mediated Mendelian Randomization Analysis
Source: Biomedicines. 2024 Aug 16;12(8):1874. doi: 10.3390/biomedicines12081874 (PMC11351342; doi:10.3390/biomedicines12081874)
Supplement: Supplementary file 1 [file biomedicines-12-01874-s001.zip › Supplementary Figure/Figure S4/Singe SNP plot of MR analysis for TL to ubm-b-1965.pdf]

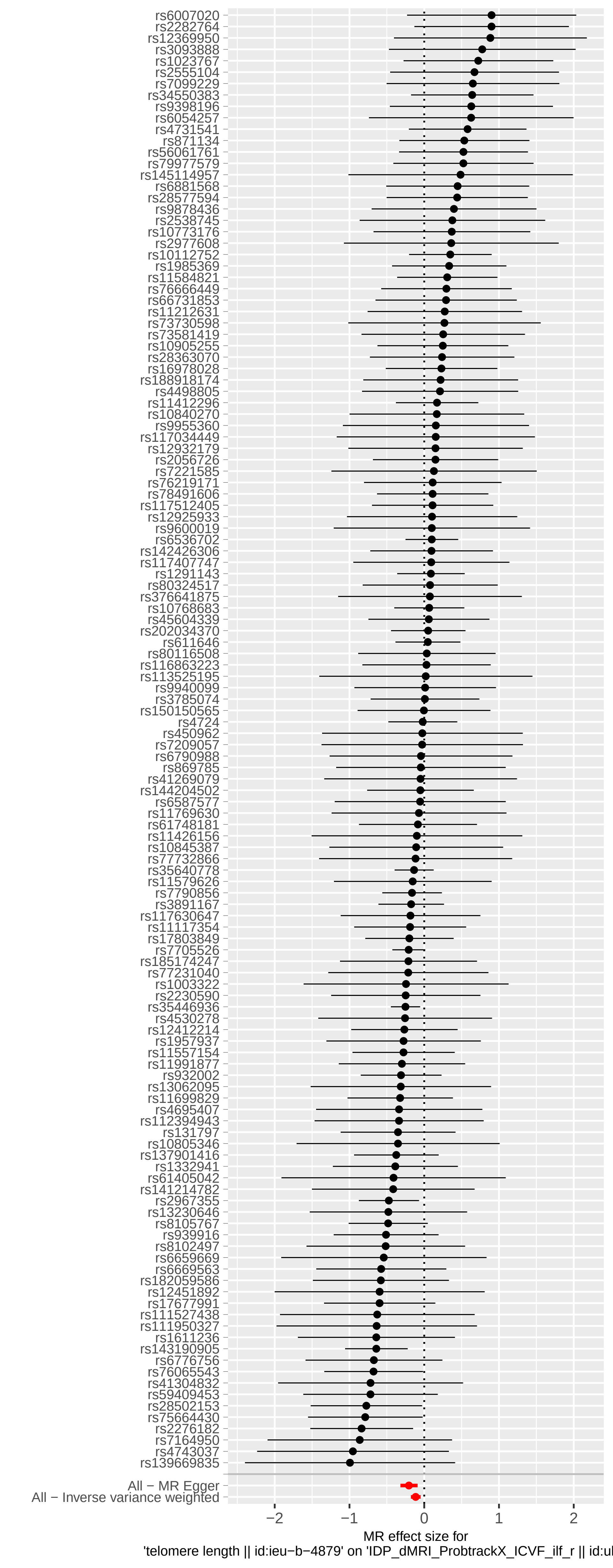
rs6007020
rs2282764
rs12369950
rs3093888
rs1023767
rs2555104
rs7099229
rs34550383
rs9398196
rs6054257
rs4731541
rs871134
rs56061761
rs79977579
rs145114957
rs6881568
rs28577594
rs9878436
rs2538745
rs10773176
rs2977608
rs10112752
rs1985369
rs11584821
rs76666449
rs66731853
rs11212631
rs73730598
rs73581419
rs10905255
rs28363070
rs16978028
rs188918174
rs4498805
rs11412296
rs10840270
rs9955360
rs117034449
rs12932179
rs2056726
rs7221585
rs76219171
rs78491606
rs117512405
rs12925933
rs9600019
rs6536702
rs142426306
rs117407747
rs1291143
rs80324517
rs376641875
rs10768683
rs45604339
rs202034370
rs611646
rs80116508
rs116863223
rs113525195
rs9940099
rs3785074
rs150150565
rs4724
rs450962
rs7209057
rs6790988
rs869785
rs41269079
rs144204502
rs6587577
rs11769630
rs61748181
rs11426156
rs10845387
rs77732866
rs35640778
rs11579626
rs7790856
rs3891167
rs117630647
rs11117354
rs17803849
rs7705526
rs185174247
rs77231040
rs1003322
rs2230590
rs35446936
rs4530278
rs12412214
rs1957937
rs11557154
rs11991877
rs932002
rs13062095
rs11699829
rs4695407
rs112394943
rs131797
rs10805346
rs137901416
rs1332941
rs61405042
rs141214782
rs2967355
rs13230646
rs8105767
rs939916
rs8102497
rs6659669
rs6669563
rs182059586
rs12451892
rs17677991
rs111527438
rs111950327
rs1611236
rs143190905
rs6776756
rs76065543
rs41304832
rs59409453
rs28502153
rs75664430
rs2276182
rs7164950
rs4743037
rs139669835
All − MR Egger
All − Inverse variance weighted
−2
−1
0
1
2
MR effect size for
'telomere length || id:ieu−b−4879' on 'IDP_dMRI_ProbtrackX_ICVF_ilf_r || id:ul
